# A novel swine model of ricin-induced acute respiratory distress syndrome

**DOI:** 10.1242/dmm.027847

**Published:** 2017-02-01

**Authors:** Shahaf Katalan, Reut Falach, Amir Rosner, Michael Goldvaser, Tal Brosh-Nissimov, Ayana Dvir, Avi Mizrachi, Orr Goren, Barak Cohen, Yoav Gal, Anita Sapoznikov, Sharon Ehrlich, Tamar Sabo, Chanoch Kronman

**Affiliations:** 1 Department of Pharmacology, Israel Institute for Biological Research, 7410001 Ness-Ziona, Israel; 2 Department of Biochemistry and Molecular Genetics, Israel Institute for Biological Research, 7410001 Ness-Ziona, Israel; 3 Veterinary Center for Preclinical Research, Israel Institute for Biological Research, 7410001 Ness-Ziona, Israel; 4 Department of Organic Chemistry, Israel Institute for Biological Research, 7410001 Ness-Ziona, Israel; 5 Infectious Disease Unit, Sheba Medical Center, 5262160 Tel-Hashomer, Israel; 6 General Intensive Care Unit, Asaf Harofeh Medical Center, 70300 Zerifin, Israel; 7 General Intensive Care Unit, Kaplan Medical Center, 7661041 Rehovot, Israel; 8 Anesthesia, Pain and Intensive Care Division, Tel-Aviv Medical Center, Tel-Aviv University, 6093000 Tel-Aviv, Israel

**Keywords:** Ricin, ARDS, Pig, Animal model, Lung permeability, Inflammation

## Abstract

Pulmonary exposure to the plant toxin ricin leads to respiratory insufficiency and death. To date, in-depth study of acute respiratory distress syndrome (ARDS) following pulmonary exposure to toxins is hampered by the lack of an appropriate animal model. To this end, we established the pig as a large animal model for the comprehensive study of the multifarious clinical manifestations of pulmonary ricinosis. Here, we report for the first time, the monitoring of barometric whole body plethysmography for pulmonary function tests in non-anesthetized ricin-treated pigs. Up to 30 h post-exposure, as a result of progressing hypoxemia and to prevent carbon dioxide retention, animals exhibited a compensatory response of elevation in minute volume, attributed mainly to a large elevation in respiratory rate with minimal response in tidal volume. This response was followed by decompensation, manifested by a decrease in minute volume and severe hypoxemia, refractory to oxygen treatment. Radiological evaluation revealed evidence of early diffuse bilateral pulmonary infiltrates while hemodynamic parameters remained unchanged, excluding cardiac failure as an explanation for respiratory insufficiency. Ricin-intoxicated pigs suffered from increased lung permeability accompanied by cytokine storming. Histological studies revealed lung tissue insults that accumulated over time and led to diffuse alveolar damage. Charting the decline in PaO2/FiO2 ratio in a mechanically ventilated pig confirmed that ricin-induced respiratory damage complies with the accepted diagnostic criteria for ARDS. The establishment of this animal model of pulmonary ricinosis should help in the pursuit of efficient medical countermeasures specifically tailored to deal with the respiratory deficiencies stemming from ricin-induced ARDS.

## Abstract (precis)

**Summary:** Lung injury induced by intratracheal instillation of ricin to swine exhibits the full set of parameters defining ARDS.

## INTRODUCTION

We report the establishment of a swine model for the in-depth study of the pathological effects of pulmonary exposure to lethal doses of ricin, a plant toxin derived from the seeds of *Ricinus communis*. In mice, the clinical pathology following pulmonary exposure to this toxin is characterized by massive influx of inflammatory cells to the lungs, histological injury of the tissue, disruption of the alveolar-capillary barrier and the generation of severe lung edema, the latter leading to respiratory failure and death (Gal et al., 2014; Sabo et al., 2015). Treatment with anti-ricin antibodies was shown to confer protection to mice following pulmonary exposure to a lethal dose of ricin (Gal et al., 2014; Sabo et al., 2015); however, survival rates were found to correlate inversely with the span of time between exposure and treatment, rendering the antibody treatment in itself of limited effectiveness when applied late after exposure. Further studies demonstrated that improved survival levels could be attained by applying an integrative therapy comprising anti-toxin antibodies together with an anti-inflammatory agent, such as a steroid compound or doxycycline (Gal et al., 2014).

Research aimed to evaluate new therapeutic options judiciously tailored for effective treatment of ricin-induced pulmonary intoxication, requires the availability of an animal model that lends itself to continuous monitoring of respiratory and hemodynamic parameters. Although recent reports have shown that a mouse model can be utilized for assessment of pulmonary gas exchange and respiratory mechanics following controlled induction of acute respiratory distress syndrome (ARDS; Patel et al., 2012, Voelker et al., 2014), mechanical ventilation and repeated blood sampling could be carried out in mice for only a very short time. These mice model systems are therefore not amenable with the surveillance of progression of ricin pathology where clinical symptoms can be discerned only many hours after exposure to the toxin and death occurs only within several days. Large animal models are surmised to have greater translational potential because of the ability to determine gas exchange performance, systemic hemodynamics and pulmonary function tests (PFTs), as well as ventilation/perfusion mismatch, over extended periods of time.

Pigs are similar to humans in terms of anatomy, genetics and physiology (Meurens et al., 2012). As in humans, and in contrast to mice, the porcine lung has extensive inter- and intra-lobular connective tissue, which connects the major vessels and the bronchi to the pleural surface (McLaughlin et al., 1961). The swine lung is considered an excellent model and has served to study lung development (Glenny et al., 2007), reperfusion injury (Budas et al., 2007) and hyperoxia-induced acute lung injury (Gushima et al., 2001), as well as other diseases. A recent study utilized the pig as a model for investigating the role of neutrophil serine proteases in human inflammatory lung diseases (Bréa et al., 2012).

Here, we delineate for the first time, the pathophysiological profile following pulmonary exposure of pigs to the ricin toxin. Lung radiographic assessment, plethysmographic monitoring, blood-gas analysis, and measurement of proinflammatory and permeability markers allowed us to determine that ricin-intoxicated pigs suffer a severe bilateral pulmonary edematous inflammation, accompanied by cytokine storming and increased permeability of the lungs. By further monitoring the progression of injury histologically, we could delineate the progression of diffuse alveolar damage (DAD) over time. Finally, by monitoring changes in the PaO_2_/FiO_2_ ratios in a mechanically ventilated ricin-intoxicated pig, we could chart the respiratory deterioration of the intoxicated animal to a stage of severe ARDS, which eventually led to respiratory collapse and death.

## RESULTS

### Pathogenesis of pulmonary ricin intoxication in pigs

Intratracheal instillation of a lethal dose of crude ricin to pigs (3 µg/kg body weight, *n*=8), resulted in the death of all the animals within a time range of 30-70 h. Following a 16-24 h asymptomatic period, visible signs of intoxication were discerned, including reduced motor activity, coughing, repeated bouts of vomiting, dyspnea and cyanosis of body extremes. Exposure of animals to a ∼3-fold higher dose of crude ricin (10 µg/kg body weight, *n*=2), accelerated the onset of the physical symptoms, and death occurred in less than 40 h. Conversely, pigs did not succumb to a 3-fold lower dose of crude ricin (1 µg/kg body weight, *n*=2), nor did they exhibit any of the above symptoms.

**Fig. 1.**
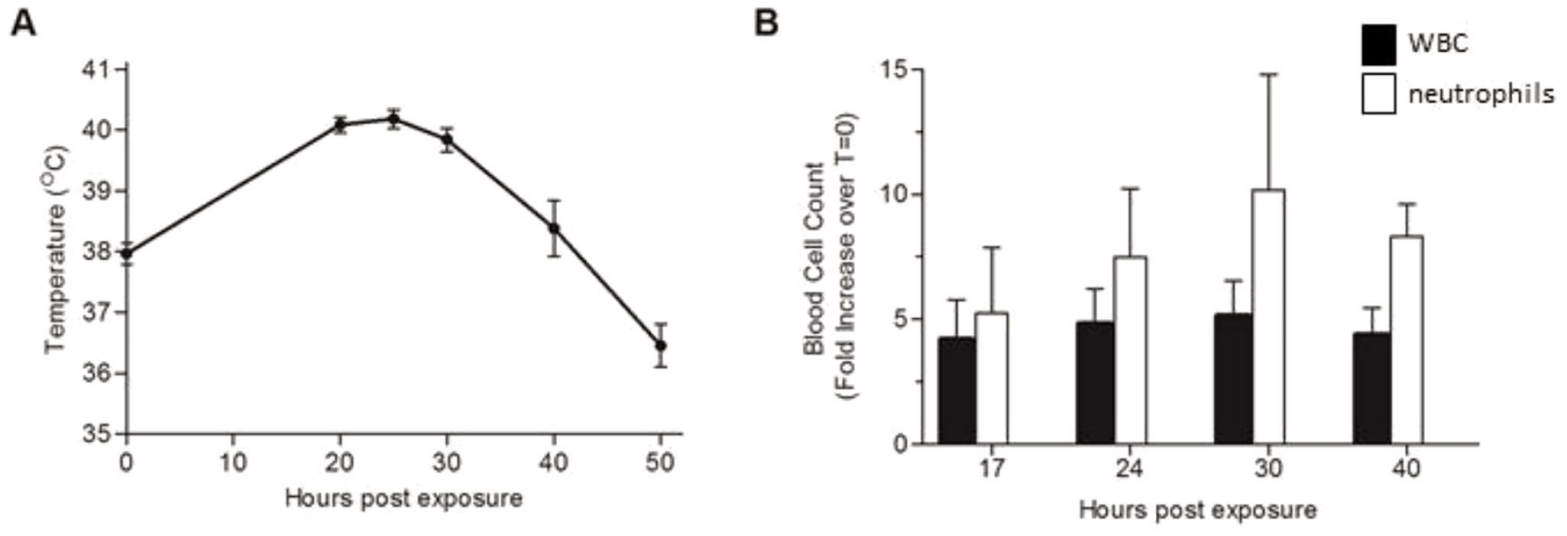
**Effect of ricin intoxication on body temperature and differential blood cell counts.** Ricin (3 µg/kg body weight) was intratracheally instilled to pigs, and at the indicated time points body temperature was measured (A) and blood samples were collected and subjected to differential count (B). White blood cell and neutrophil values are expressed as fold increase over pre-exposure level (*t*=0, measured prior to intoxication) determined for each subject. Values are means±s.e.m., *n*=5.

To allow full characterization of the ricin-induced pathology, groups of 4-6 pigs were intratracheally exposed to the lethal dose of ricin and various physiological parameters were scrutinized. Monitoring body temperature of the intoxicated pigs revealed that during the first 24 h temperatures rose from 38±0.4°C to 40±0.3°C (Fig. 1A). Temperatures declined to normal at around 40 h post-exposure (hpe) and continued to fall to 36.5±0.5°C at 50 hpe.

Hematological analysis of blood samples from ricin-intoxicated pigs displayed an increase over time in white blood cell counts in general and in neutrophil counts in particular; neutrophil counts in the circulation at 17 and 30 hpe were around 5- and 10-fold higher than counts at baseline, respectively (Fig. 1B).

**Fig. 2.**
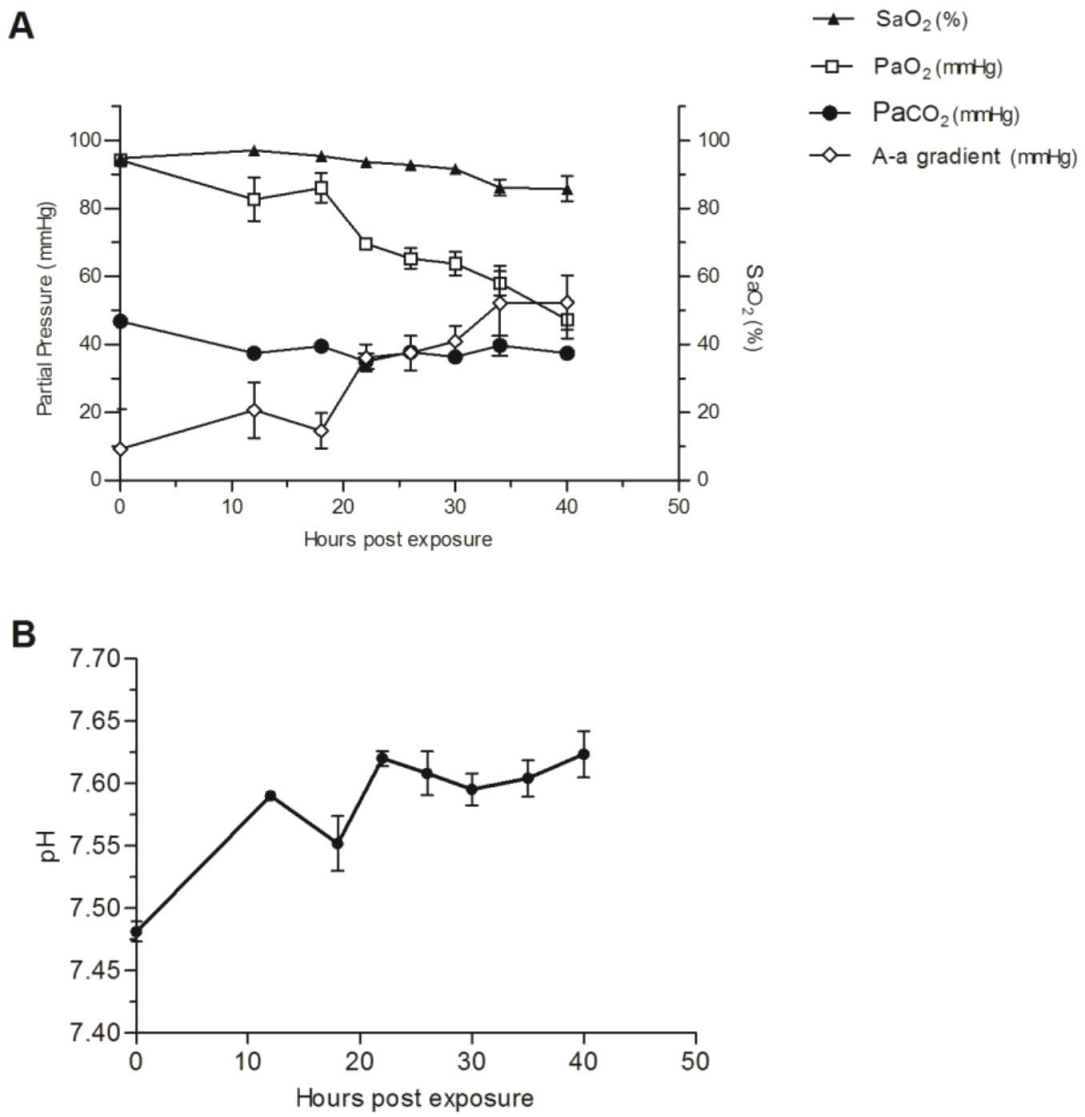
**Changes in blood gas exchange and pH following ricin intoxication.** Ricin (3 µg/kg body weight) was intratracheally instilled to pigs, arterial blood samples were withdrawn at 0, 12, 18, 22, 26, 30, 34 and 40 hpe and (A) SaO_2_, PaO_2_, PaCO_2_, A-a gradient and (B) pH values were determined. All pH values measured were significantly different from *t*=0 (*P*<0.05). *t*=0 values were determined prior to intoxication. Values are means±s.e.m., *n*=5.

To study the respiratory pathophysiological changes ensuing ricin intoxication, two sets of experiments were performed: gas exchange measurements were taken from arterially cannulated pigs and respiratory dynamics were determined by barometric whole-body plethysmography (bWBP) in non-anesthetized pigs. It is evident from arterial blood gas analysis that the animals experienced acute hypoxemia. Specifically, arterial partial pressure of oxygen (PaO_2_) declined over time, reaching a value of ∼50 mm Hg at 40 hpe (Fig. 2A) by which time oxygen saturation values (SaO_2_) also dropped to 85%. To assess the source of hypoxemia, the alveolar-arterial O_2_ difference (PAO_2_-PaO_2_), also named the A-a gradient, was calculated from the alveolar gas equation: PAO_2_=FiO_2_(Patm-PH_2_O)–PaCO_2_/R, where FiO_2_ (the fractional concentration of inspired O_2_ when breathing room air) is 0.21; Patm (barometric pressure at sea level) is 760 mm Hg, PH_2_O (water vapor pressure when air is fully saturated at 37°C) is 47 mm Hg; PaCO_2_ is the arterial partial pressure of carbon dioxide; and R (respiratory quotient or ratio of CO_2_ production to O_2_ consumption) is 0.8. In ambient conditions, PAO_2_ is equal to 150−1.25×PaCO_2_, which in our case was ∼100 mm Hg throughout the study since PaCO_2_ remained relatively constant (∼40 mm Hg). The calculated A-a gradient was pathological as early as 12 hpe (20 mm Hg; normal values <15 mm Hg) and kept elevating to highly abnormal values, reaching 50 mm Hg at 40 hpe (Fig. 2A). This increased A-a gradient could be explained by a defect in diffusion, ventilation perfusion mismatch or right-to-left shunt (Rodríguez-Roisin and Roca, 2005). The inability to correct the low PaO_2_ with supplementary oxygen (see below) suggests shunting as the cause of hypoxemia.

**Fig. 3.**
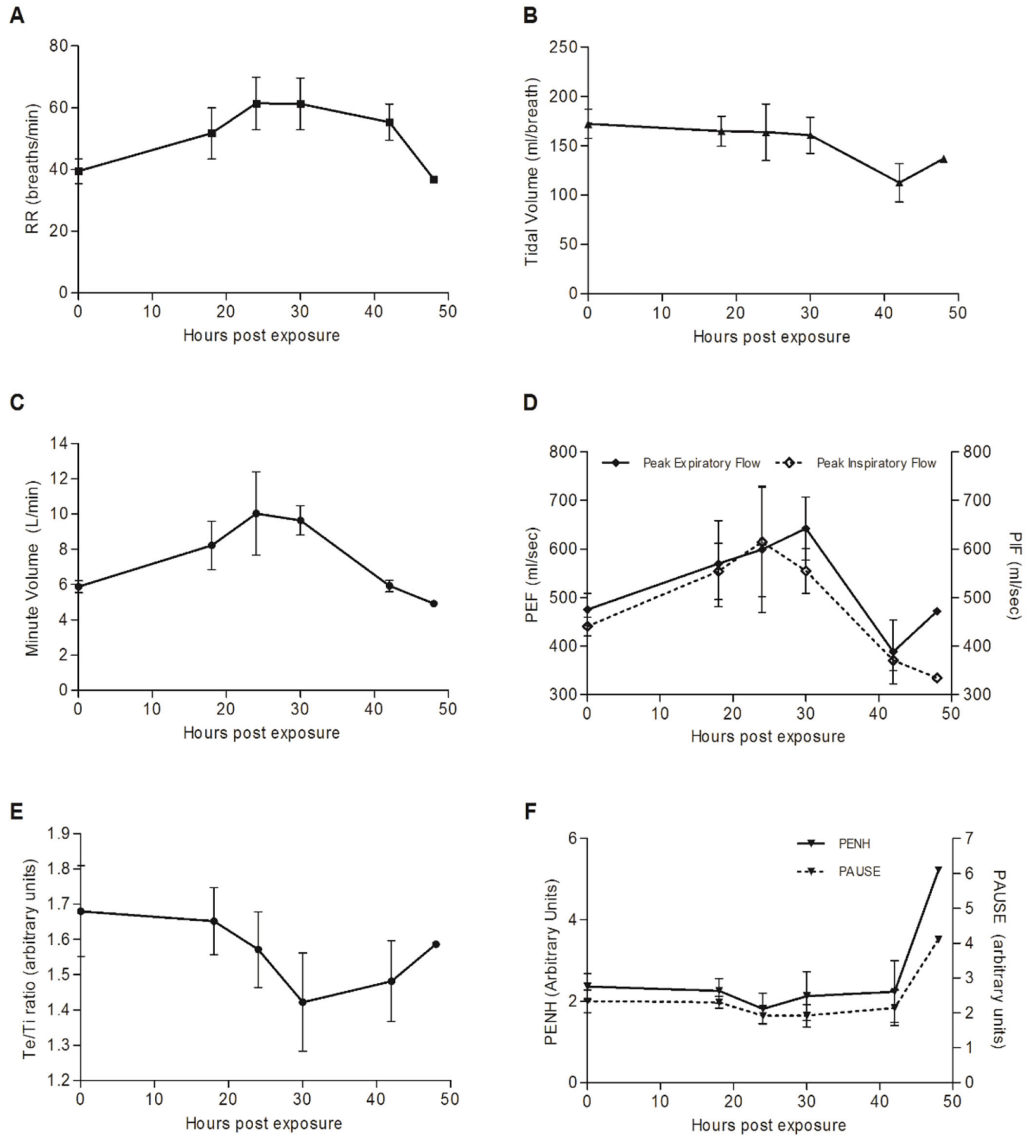
**Pulmonary function tests (PFTs) in ricin-intoxicated pigs.** Following intratracheal instillation of ricin (3 µg/kg body weight), respiratory dynamics were measured in non-anesthetized pigs at 0, 18, 24, 30, 42 and 48 hpe by barometric whole-body plethysmography. Control plethysmographic values were determined individually for each of the subjects, prior to intoxication. (A) Respiratory rate (RR). (B) Tidal volume. (C) Minute volume. (D) Peak inspiratory and expiratory flow. (E) Ratio of expiratory time to inspiratory time (E:I). (F) PENH and pause. Values are means±s.e.m., *n*=5.

As mentioned above, to further investigate and document respiratory dynamics following ricin intoxication, five pigs were monitored at several time points by bWBP. Control plethysmographic values were determined individually for each of the pigs prior to intoxication. As early as 18 hpe, respiratory rates were elevated by 30% in comparison to control values and kept rising: to 150% of control level by 24 hpe (Fig. 3A). Examination of tidal volume (TV) over time demonstrated that it did not alter significantly up to 30 hpe (Fig. 3B) and only at later time points did levels decline markedly. Minute volume (MV), a product of respiratory rate and tidal volume, rose to 140% of control values at 24-30 hpe, mainly due to an increase in RR and not in TV (Fig. 3C). The fact that the measured values of PaCO_2_ remained stable throughout the entire time span of the experiment (Fig. 2A), together with the elevation of both the A-a gradient and MV, means that extraordinary respiratory effort was invested and that, at least until 30 hpe, lung ventilation was not compromised. This reaction of elevation in MV (mainly by increasing RR and not TV) is a normal physiological response of healthy and strong animals with good lung reserve to mounting hypoxia. At later time points, decompensation occurs and MV values fall, so that by 40 hpe, although they are similar to baseline levels, they are clearly insufficient to maintain satisfactory tissue oxygenation (see Fig. 2A). Animals exhibited slight but significant (*P*<0.05 at all time points) alkalosis under normal PaCO_2_ levels (Fig. 2B), as manifested by an increase in the pH values of blood from 7.48±0.02 to 7.62±0.03. This might signify a mixed acid-base disorder, which is most probably a result of vomiting and hyperventilation.

The fact that Pause, an indicator of assumed bronchoconstriction and PENH (enhanced pause), a putative measurement of lung resistance (Rezk et al., 2008), did not alter significantly throughout the study (Fig. 3F) most likely indicates that the observed elevation in respiratory effort was not due to overcoming any resistance to airflow. Although the diagnostic specificity of this endpoint (Pause and PENH) has been questioned (Bates et al., 2004), absence of airflow obstruction is further supported by elevation in peak inspiratory and expiratory flows (Fig. 3D), along with a relatively stable E:I ratio [ratio of the duration of expiration (Te) to the duration of inspiration (Ti)] values over time (Fig. 3E). Elevation in the respiratory effort with no evidence of airflow obstruction suggests a pathological progressing process in the lung parenchyma (Naureckas and Solway, 2012), in our case, an alveolar-filling disease due to ricin intoxication.

**Fig. 4.**
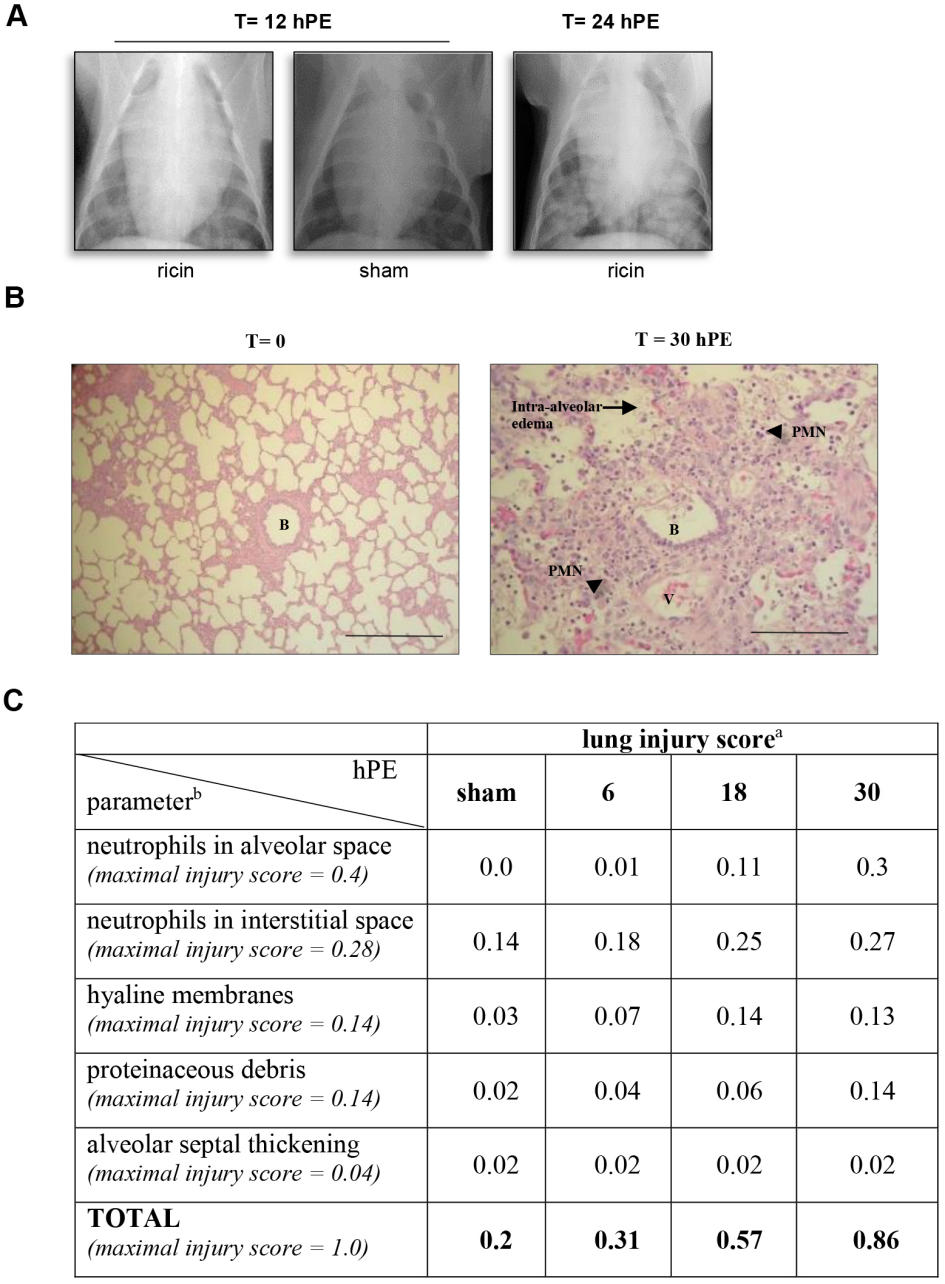
**Lung injury following ricin intoxication.** (A) X-ray. CXR radiographs were taken at 12 and 24 hpe to ricin (3 µg/kg body weight) and representative CXRs are shown. *t*=12 hpe ricin: representative CXR (1 of 3) of ricin-intoxicated pig at 12 hpe; *t*=24 hpe ricin: representative CXR (1 of 3) of ricin-intoxicated pig at 24 hpe; *t*=12 hpe, sham: CXR radiograph taken 12 h after sham intoxication. (B) H&E-stained lung section of a ricin-intoxicated pig. Lungs were removed at 30 hpe to ricin (3 µg/kg body weight) and subjected to histological analysis (400×). B, bronchiole; V, blood vessel; arrow, intra-alveolar edema; arrowheads, PMNs. Scale bars: 100 µm. (C) Lung injury scoring following ricin intoxication. ^a^Lung injury score was determined by scanning ≥20 random high-power fields (×400) of both lungs of two pigs per time point. ^b^Maximal injury scores of the various parameters were assigned by the American Thoracic Society Committee (Matute-Bello et al., 2011) according to their relevance to experimental acute lung injury. *n*=8.

Repeated radiographic assessment of lung injury (chest X-ray, CXR) demonstrated the presence of bilateral opacities as early as 12 hpe, while at 24 hpe, diffuse bilateral infiltrations were observed throughout the lungs (Fig. 4A). A sham animal intratracheally instilled with diluent at the same volume as that instilled to intoxicated animals displayed no X-ray pathology (Fig. 4A). Interestingly, although there is clear evidence of pulmonary pathology on CXR at 12 hpe, this pathology could not be discerned at that time point by any of the other physiological parameters that we monitored.

To determine any ricin-inflicted injury at the tissue level, Hematoxylin and Eosin (H&E)-stained paraffin sections prepared from various organs of intoxicated pigs at 30 hpe, were analyzed. Histological injury was confined to the lungs and was not observed in the liver, spleen, kidney, adrenal, pancreas or heart (data not shown). The salient pathological findings in the pig lungs included sequestration of PMNs in the alveolar spaces and interstitium, hemorrhage, interstitial and intra-alveolar edema, thickening of alveolar septa, as well as the occasional formation of hyaline membranes, all of which are key features of diffuse alveolar damage (DAD, Fig. 4B).

Quantification of histological lung injury was determined by applying a system recently suggested (Matute-Bello et al., 2011). This scoring system, which takes into account neutrophils in alveolar and interstitial spaces, proteinaceous debris filling the airspaces, septal thickening of the alveolar wall and the presence of hyaline membranes, was applied by scanning at least 20 random high-power fields (×400) of both lungs of two intoxicated pigs for each time point. Sham-intoxicated pigs were also scanned. Using this method, at the late time point of 30 hpe, a score of 0.86 out of a maximal injury score of 1, was determined (Fig. 4C). To appreciate the development of tissue injury over time, scoring was also performed of lung specimens prepared at earlier time points. Injury scores at 6 and 18 hpe were 0.31 and 0.57, respectively. The increase in score between 18 and 30 hpe reflects an increase in the levels of 2 out of the 5 criteria: neutrophils in the alveolar space and the presence of proteinaceous debris. Neutrophils in the interstitial space as well as the presence of hyaline membranes, reached their maximal levels at 18 hpe. Although septal thickening was noticed in some fields, the scoring of this variable even at late time points following ricin intoxication did not exceed the score determined for sham-intoxicated pig samples.

### Pulmonary inflammation following ricin intoxication

**Fig. 5.**
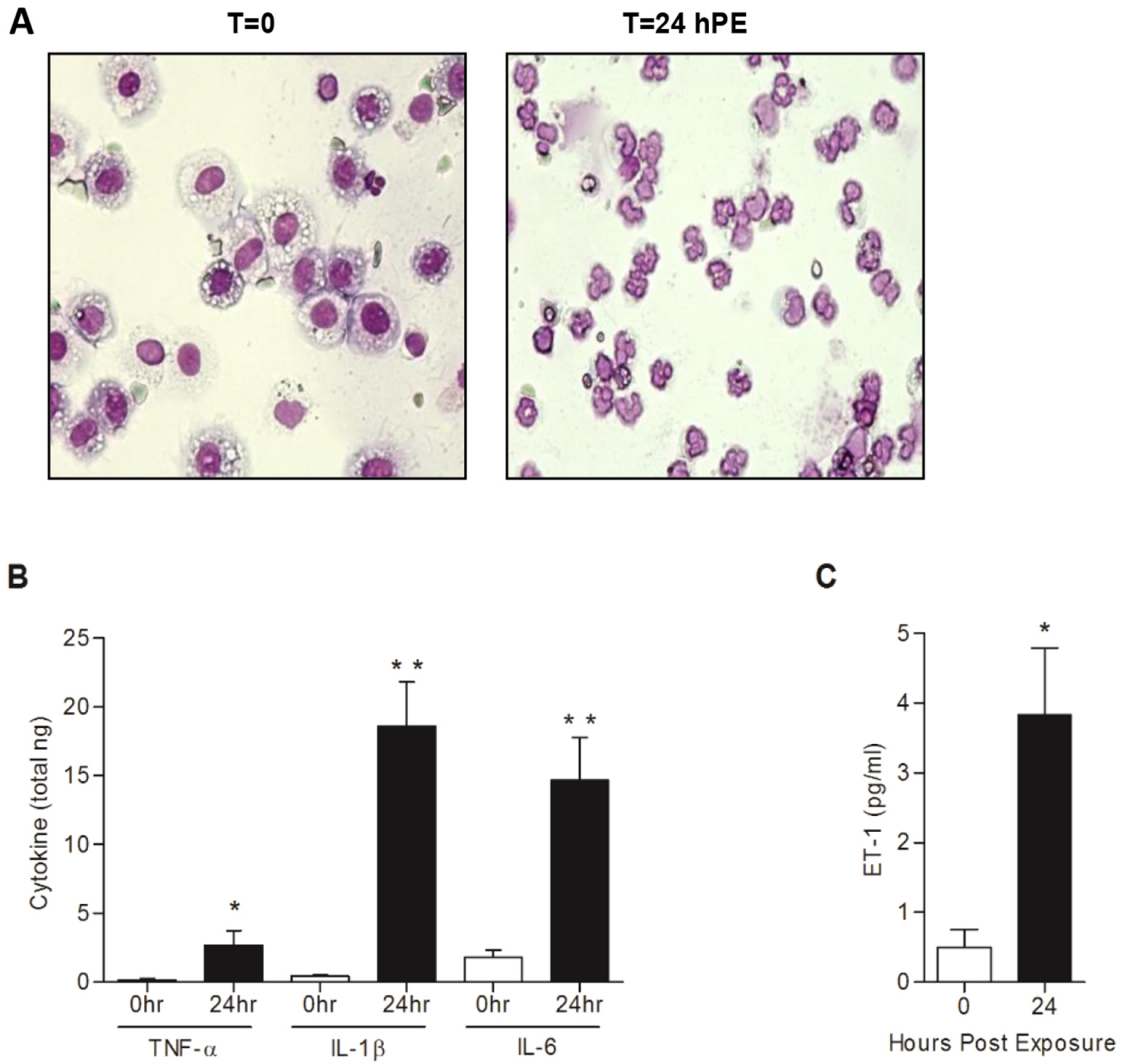
**Changes in lung cell composition, proinflammatory cytokines and ET-1 in the BALF of ricin-intoxicated pigs.** Bronchioalveolar lavage was performed before or 24 hpe to ricin (3 µg/kg body weight). (A) Cells harvested from the BALF of a representative pig. Note that >99% of the cells at *t*=0 and at *t*=24 hpe, are of macrophage and polymorphonuclear origin, respectively. (B) Concentration of the pro-inflammatory cytokines TNF-α, IL-1β and IL-6 in the BALF. (C) Concentration of the peptide vasoconstrictor ET-1 in the BALF. Values are means±s.e.m., *n*=5. *P<0.05; **P<0.005.

Profusion of neutrophils in the lungs is a well-documented feature of pulmonary inflammation. Bronchioalveolar lavage (BAL) of pig lungs was performed immediately before exposure to ricin and then again at 24 hpe. At *t*=0, total bronchioalveolar lavage fluid (BALF) cell counts were 8×10^6^ (±1×10^6^), of which >99% were macrophages. At 24 hpe, elevated cell counts of 50×10^6^ (±6×10^6^) were observed, polymorphonuclear cells accounting for nearly all (>99%) of the cells in the BALF collected at this time point (Fig. 5A). In addition, the levels of the pro-inflammatory cytokines TNF-α, IL-1β and IL-6 in the BALF collected from intoxicated swine at 24 hpe, were found to be raised significantly to 8- to 45-times baseline levels (Fig. 5B).

### Alterations of the alveolar capillary barrier

Increase in lung wet weight is an indicator of alveolar-capillary barrier disruption and can serve as a measure of extravascular lung water (EVLW; Grainge et al., 2010). To appraise the deleterious effect exerted by intratracheal ricin exposure on alveolar permeability, changes in the ratio of lung-wet-weight (LWW) to body-weight (BW) were monitored. To this end, the relative weights of lungs of ricin-intoxicated pigs harvested at 24 hpe were compared with those of naïve pigs (Table 1). Indeed, LWW/BW (g/kg) in the intoxicated pigs (24.6±1.5) was more than double the level measured in naïve pigs (10.0±0.48). Augmentation of alveolar permeability following exposure to ricin was also shown by the considerable increase in total protein content in the BALF. While BALF of naïve pigs was characterized by protein levels of 1.2±0.6 mg/lung, protein measurements in the BALF collected from ricin-intoxicated pigs were significantly higher, reaching 118±84 mg/lung at 24 hpe.

**Table 1.**
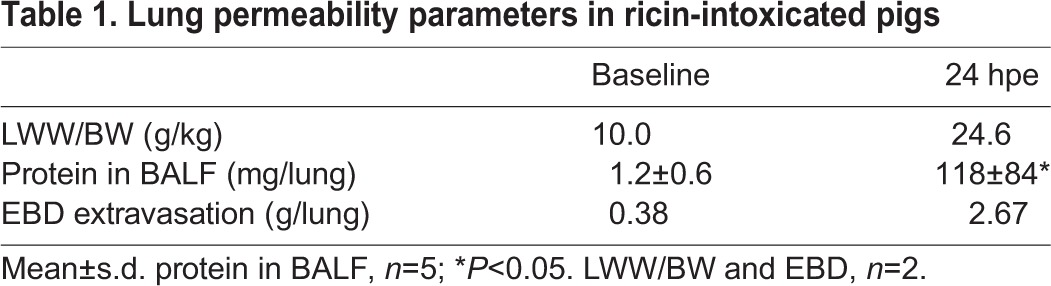
**Lung permeability parameters in ricin-intoxicated pigs**

To appreciate the magnitude of alveolar-capillary barrier dysfunction following ricin intoxication we performed an Evans Blue dye (EBD) assay. Following intravenous injection of EBD, the dye binds to circulating albumin and extravasates to the lungs as an albumin-dye complex only if this organ is permeable at the time of injection. Pulmonary EBD values in 24 hpe ricin-intoxicated pigs were found to be ∼7-fold higher than in the naïve animals (Table 1). These values reflect the magnitude of influx of albumin into the lungs within the short period of time that elapsed between EBD injection and the harvesting of the lungs (15 min).

Finally, since elevated levels of endothelin-1 (ET-1 also known as EDN1), a potent peptide vasoconstrictor, were shown to correlate with increased pulmonary water contents (Kuzkov et al., 2006), we examined whether pulmonary ricin intoxication promotes high levels of ET-1 expression (Fig. 5C). Indeed, 24 h after ricin exposure, ET-1 levels in the BALF were found to be ∼8-fold higher than the ET-1 levels determined in the BALF of pigs prior to exposure.

### Physiological dysfunction following ricin intoxication

ARDS in humans is defined by a set of clinical parameters (ARDS Definition Task Force, 2012, Matute-Bello, et al., 2011). As detailed above, radiological evidence of diffuse bilateral pulmonary infiltrates, which serve as one of these parameters, was clearly evident in pigs following intratracheal exposure to a lethal dose of ricin. Other parameters pertinent to ARDS diagnosis include appearance within no more than a week from the suspected insult, the absence of clinical evidence of elevated left atrial hypertension and a ratio of the partial pressure of arterial oxygen to the fraction of inspired oxygen (PaO_2_/FiO_2_) of less than 300.

To determine whether the pulmonary pathology in ricin-intoxicated pigs complies with these parameters as well, we first determined that the respiratory failure following ricin intoxication, evident from the plethysmographic and blood-gas indices, is not a result of cardiac failure. To this end, various cardiovascular parameters including invasive blood pressure (exhibited as mean arterial pressure, MAP), heart rate (HR) and non-invasive cardiac output (CO) were measured in five arterially cannulated ricin-intoxicated pigs at 24 hpe (Table 2). Determination of these parameters in each of the pigs prior to intoxication, served as the baseline.

**Table 2.**
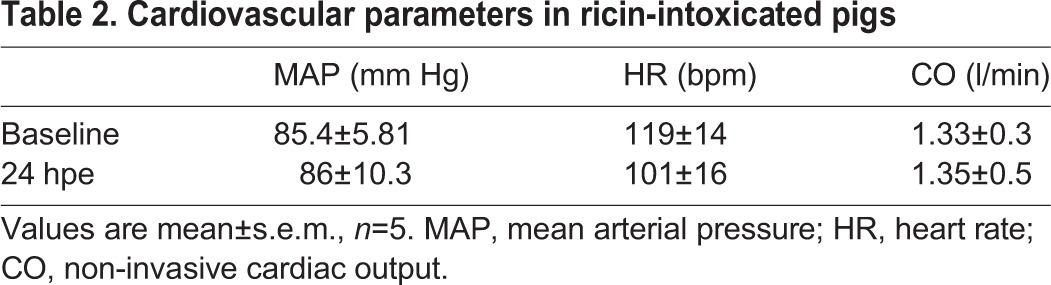
**Cardiovascular parameters in ricin-intoxicated pigs**

All of the hemodynamic parameters determined at 24 hpe were not significantly different from the corresponding baseline values. In addition, when the 5 pigs were monitored by 3-lead ECG, the ECG recordings showed normal sinus rhythm with no signs of ischemia (data not shown). Collectively, these findings clearly demonstrate that the documented respiratory failure following exposure to ricin is not secondary to cardiac failure.

**Fig. 6.**
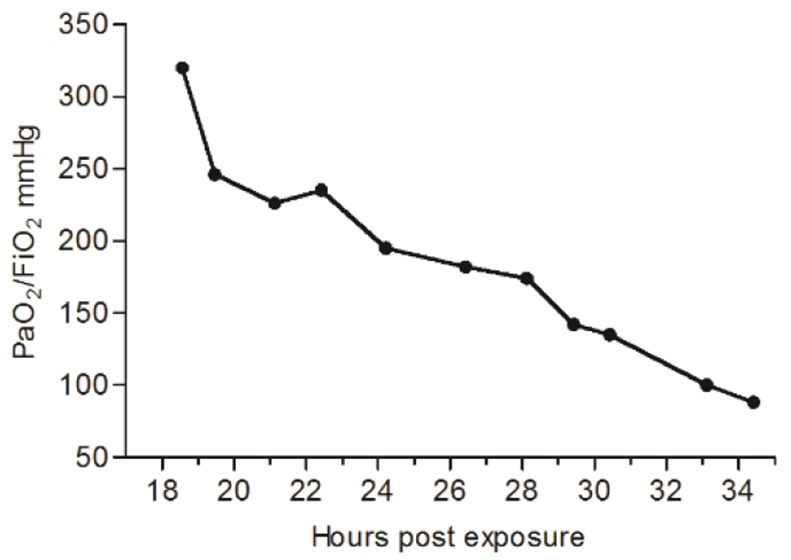
**Changes in PaO_2_/FiO_2_ index following ricin intoxication.** PaO_2_/FiO_2_ ratios were calculated at the indicated time points in a mechanically ventilated ricin-intoxicated pig (*n*=1).

Lastly, PaO_2_/FiO_2_ values were determined in a ricin-intoxicated pig. To this end an arterially cannulated intoxicated pig was mechanically ventilated from 18 hpe onwards. Arterial blood samples collected between 19 hpe and the time of death (35 hpe) were promptly subjected to gas exchange measurements and PaO_2_/FiO_2_ values were calculated (Fig. 6). Close to the onset of ventilation, a PaO_2_/FiO_2_ value of 320 was documented, attesting to the fact that at this stage the pig did not develop ARDS. However, a sharp decline in physiological function was noted over time. Mild ARDS (200 mg Hg<PaO_2_/FiO_2_≤300) observed at 20-23 hpe was followed by moderate ARDS (100 mg Hg<PaO_2_/FiO_2_≤200) at 24-33 hpe. The last blood-gas sampling taken at 34.5 hpe gave rise to a PaO_2_/FiO_2_ of 88, indicative of severe ARDS (PaO_2_/FiO_2_<100 mg Hg).

## DISCUSSION

Intensive studies of the pathogenesis and treatment of acute lung injury have been carried out several years and much is known about the different insults which can induce ARDS (Matthay and Zemans, 2011). Animal experimental models, pivotal for the exploration of pathogenesis and for evaluation of novel treatment approaches to ARDS, have contributed significantly to the development of medical countermeasures against this life-threatening syndrome (Wang et al., 2008; Bastarache and Blackwell, 2009; Ballard-Croft et al., 2012). Ricin, a plant toxic protein readily produced from the seeds of *Ricinus communis* (castor beans), is considered to be a bio-terrorism threat of concern, particularly via the inhalation route of exposure (Lindsay, 2015). Ricin exerts its cytotoxic effect by site-specific depurination of adenine 4324 of the 28S rRNA in the 60S ribosomal subunit. This irreversible impairment of the ribosome prevents binding of elongation factor-2, leading, in turn, to protein synthesis arrest and cell death (Audi et al., 2005). Studies carried out at our laboratory with the mouse animal model demonstrated that pulmonary exposure to ricin resulted in severe neutrophilic lung inflammation accompanied by an unbridled cytokine storm and extensive damage of the lung tissue (Gal et al., 2014; Sapoznikov et al., 2015). Several mechanisms were proposed (Korcheva et al., 2005, 2007; Lindauer et al., 2010) to explain possible causal relationships between the molecular enzymatic activity of ricin (i.e. 28S rRNA depurination) and the clinical manifestations of ricin intoxication (such as the onset of an edematous neutrophilic lung inflammation following pulmonary exposure), but these issues have yet to be resolved. Irrespective of the exact mechanism involved, the onset of a ricin-induced rampant inflammatory response in the lungs could rapidly deteriorate to neutrophil-dependent impairment of the alveolar-capillary barrier and subsequent formation of a severe life-threatening edema.

Comprehensive assessment of the pulmonary pathology that develops in mice following ricin intoxication is limited by the fact that respiratory physiology in this animal model cannot be monitored over a sufficiently long period of time. Moreover, small animals are less than ideal models for evaluation of lung injury, owing to their small body size and variations in physiology (Jugg et al., 2011). Also, they do not reproduce salient pathologic features of acute lung injury in humans, for instance the histopathological correlate DAD (Matute-Bello et al., 2011). Several studies carried out in our laboratory as well as others, have implicated that the pathological state ensuing pulmonary exposure of experimental animals to ricin is that of ARDS (Korcheva et al., 2007; Gal et al., 2014); however, it is quite difficult to translate the full set of criteria used to define human ARDS to rodent animal models. In recent years, a number of studies provided important insights into the clinical signs and the gross and histological lesions that develop in the lungs of non-human primates following pulmonary exposure to ricin (Bhaskaran et al., 2014; Roy et al., 2012, 2015; Pincus et al., 2015) but a comprehensive study encompassing the various parameters characteristic of ARDS has not been performed in these animal models. We note that these models do not easily lend themselves to the quantitative study of the broad spectrum of pathological constituents of ricin toxicosis, since such an endeavor requires surveillance of a large number of subjects.

In the present study, we report the establishment of a pig animal model for monitoring pulmonary ricinosis. Following intratracheal instillation of ricin, the intoxicated animal fully reproduced the entire set of clinical parameters developed for defining acute lung injury and ARDS. This includes acute onset, radiological evidence of diffuse bilateral pulmonary infiltrates, PaO_2_/FiO_2_ of less than 300 and no clinical evidence of cardiac failure or fluid overload (ARDS Definition Task Force, 2012). Moreover, in our pig animal model, ancillary markers of acute lung injury such as histological evidence of tissue injury, permeabilization of the alveolar-capillary barrier and evidence of an inflammatory response, could also be readily documented.

Following intratracheal exposure to a lethal dose of ricin, the intoxicated pigs displayed a transient elevation of body temperatures, which then declined to lower than normal values. In previous studies carried out in the mouse model at our laboratory, body temperature declined steadily from the time of exposure onward and fever could not be observed (data not shown). The disparity between the two animal models is in line with previous reports, finding that pigs, in a manner similar to humans, display a hyperthermal response to endotoxin, unlike mice, which are relatively resistant to endotoxin shock and display hypothermia (Fairbairn et al., 2011).

BALF analysis performed in the pigs at 24 hpe demonstrated that a massive recruitment of neutrophils to the lungs has occurred. At this time point, elevated levels of TNF-α, IL1β and IL-6 were also noted. The presence of elevated levels of these three pro-inflammatory cytokines in the BALF of pigs following induction of acute respiratory distress by repeated saline lavage has been documented previously (Muellenbach et al., 2009).

High levels of the vasoconstrictor peptide ET-1, were also measured in the BALF of the ricin-intoxicated pigs. ET-1, the most abundant isoform of the endothelin peptide family, is produced by a variety of cells, including airway epithelium and alveolar epithelial cells (Blouquit et al., 2003; Jain et al., 2007; Comellas et al., 2009) and it is known to be released in response to various pathological states, including high level expression of pro-inflammatory cytokines (Marasciulo et al., 2006; Frank et al., 2007). Elevated levels of circulating ET-1 are considered to be a marker for endothelial dysfunction (Nakano et al., 2007), and individuals with an elevated EVLW index have significantly raised ET-1 levels (Kuzkov et al., 2006). Indeed, in the present study the ricin-intoxicated pigs exhibited a prominent increase in extravascular lung water, as reflected by a greater than 2-fold increase in the LWW/BW ratio. We note that increased EVLW is at the center of the pathophysiologic changes in ARDS and there is a clear prognostic relationship between EVLW and lung injury (Chew, 2013; Phillips, 2013). Various mechanisms were proposed for the ET-1-induced pulmonary edema, such as disruption of the alveolar-capillary barrier due to the influx of inflammatory cells or upregulation of mediators that promote vascular permeability (Kosmidou et al., 2008). Recent studies suggested that ET-1 not only induces edema build-up but also prevents edema resolution by impairing alveolar fluid clearance (Berger et al., 2009; Comellas et al., 2009).

Monitoring pulmonary ricinosis as well as other respiratory disorders in a large animal model such as the pig has clear advantages because respiratory function measurement is feasible over long periods and invasive procedures required for repeated blood-gas sampling can be readily performed. In the present study, by monitoring respiratory dynamics and functions over time by barometric whole-body plethysmography, we could ascertain that pulmonary exposure to ricin brings about a marked elevation in the effort of breathing without any apparent airflow obstruction, while repeated gas exchange analysis in arterially cannulated animals enabled us to determine that intoxicated pigs undergo acute hypoxemia with no signs of hypoventilation. Likewise, radiographic assessment and pulmonary histological examination confirmed the presence of diffuse bilateral infiltrations and diffuse alveolar damage (DAD) in the lungs, respectively. Interestingly, the radiographic changes documented in our study occurred very early, as opposed to the radiographic latent period usually observed during the first 24 h following the initial insult in most cases of indirect or nonpulmonary ARDS (e.g. sepsis or trauma; Sheard et al., 2012). This suggests that CXR may serve as an important clinical tool to evaluate the progress of ricin-induced pulmonary disease.

Finally, monitoring respiratory parameters in a mechanically ventilated pig allowed us to chart the functional deterioration of the animal through consecutive stages of increasing severity, as judged by the declining values of PaO_2_/FiO_2_, while non-invasive cardiac output monitoring of the intoxicated pigs eliminated the possibility that the ricin-intoxicated pigs suffered from cardiac failure. We note that determination of PaO_2_/FiO_2_ values were based on the surveillance of a single pig, which was mechanically ventilated from 18 hpe until death, which occurred 17 h later, and it is reasonable to assume that the transition time points through the various stages, from mild to moderate to severe ARDS, would vary in different subjects. Taken together, these data are indicative of a pathological process in the lung parenchyma that exhibits all of the previously documented traits of ARDS. It is worth noting in this context that ARDS has been intimately related to the damage of pulmonary epithelial cells in particular (Ware, 2006; Gropper and Wiener-Kronish, 2008). Indeed, studies carried out at our laboratory demonstrated that following pulmonary exposure of mice to ricin, ribosome depurination levels were inordinately higher in epithelial cells compared with other cell types populating the lung (Falach et al., 2016), whereas tracking of pulmonary cell elimination by various methods allowed us to determine that ricin-induced epithelial injury is mostly confined to a particular subset of epithelial cells – the alveolar type II cells (Sapoznikov et al., 2015).

The pathological studies of pulmonary intoxication by ricin carried out in our laboratory aimed to aid in the development of efficient therapeutic countermeasures. Studies carried out with rodent animal models allowed us to decipher important features of the pulmonary disorder induced by the toxin (Sapoznikov et al., 2015; Falach et al., 2016), as well as to evaluate post-exposure treatments based on the use of anti-ricin antibodies in conjunction with anti-inflammatory compounds (Gal et al., 2014, 2016). The establishment of a large animal model for pulmonary ricinosis that is fully compatible with on-line monitoring of respiratory performance is now expected to aid us in our search for therapeutic interventions, such as respiratory support, that are specifically tailored to deal with the well-defined respiratory shortcomings stemming from ricin-induced ARDS.

## MATERIALS AND METHODS

### Animals

All experiments were carried out in accordance with the Israeli law and were approved by the Ethics Committee for Animal Experiments at the Israel Institute for Biological Research. Treatment of animals was in accordance with regulations outlined in the USDA Animal Welfare Act and the conditions specified in the National Institute of Health Guide for Care and Use of Laboratory Animals.

Young female pigs (Topigs 20, a cross between Yorkshire female and Landrace male; 11.2-16.1 kg, age 9-11 weeks, *n*=45) were obtained from an approved commercial source (van Beek, Netherlands), fed on standard pig diet and housed in a purpose-built animal holding facility for 4-8 days prior to the start of the experiment. Animals were allowed *ad libitum* access to food; 12 h before the experimental procedure, food was withdrawn while water remained freely available. Lighting was set to mimic a 12 h:12 h light:dark cycle. Number of pigs used per experimental procedure is detailed in the figure legends.

### Ricin preparation and exposure

Crude ricin was prepared from seeds of endemic *Ricinus communis* essentially as described (Lin and Liu, 1986). Briefly, seeds were homogenized in a Waring blender in 5% acetic acid in phosphate buffer (Na_2_HPO_4_, pH 7.4). The homogenate was centrifuged and the clarified supernatant containing the toxin was subjected to ammonium sulfate precipitation (60% saturation). The precipitate was dissolved in PBS and dialyzed extensively against the same buffer. The toxin preparation appeared on a Coomassie Blue-stained non-reducing 10% polyacrylamide gel as two major bands of molecular mass ∼65 kDa (ricin toxin, ∼80%) and 120 kDa (*Ricinus communis* agglutinin, ∼20%). Protein concentration was determined as 2.86 mg/ml by 280 nm absorption (NanoDrop 2000, Thermo Fisher Scientific, Waltham, MA, USA).

For intoxication, pigs were anesthetized with intramuscular (i.m.) ketamine and xylazine (10 and 1 mg/kg, respectively) and thereafter an ear vein was punctured and intravenous anesthesia was deepened with propofol (1%, 1-2 ml), immediately followed by intubation with a cuffed endotracheal tube (5.5-6.0 mm internal diameter). Intratracheal penetration was verified by capnography and localization of the distal tube-end above the tracheal bifurcation was verified by chest X-ray. Crude ricin (3 µg/ml/kg) divided into two portions was instilled to the raised (∼30°) supine pig while tilting the animal from right to left. In preliminary experiments using a medical contrast agent (iopromide, Ultravist), X-ray analysis attested that nearly homogeneous lung distribution is achieved using this method of instillation.

### Surgical preparations

#### Cannulation preparative surgery

Experiments in which the pigs were sampled for arterial blood and for measuring invasive blood pressure, required insertion of an arterial line to the carotid artery, a procedure that was performed 3 days before intoxication. The left common carotid artery of pigs (anesthetized by intramuscular injection of ketamine and xylazine as described above, followed by inhalation of isoflurane 2-4% with 100% oxygen) was surgically exposed under aseptic conditions and a single lumen catheter (Biometrix; 16 Ga×20 cm, Jerusalem, Israel) was introduced. Cannulated pigs were administered cephalosporin (Ceforal, 30 mg/kg; Teva, Petah-Tiqva, Israel) once a day for 3 days.

#### Mechanical ventilation

Carotid arterial as well as multi-lumen jugular venal (Biometrix; 8.5 Fr×20 cm, Jerusalem, Israel) cannulations were performed 3 days before intoxication, as above. At 18 hpe, a clinically relevant time point for medical intervention and respiratory surveillance, the pig was anesthetized by intramuscular injection of ketamine and xylazine (see above) and mechanical ventilation was performed using a ventilator (Hamilton C1, Hamilton Medical, Bonaduz Switzerland). Anesthesia was maintained with propofol and fentanyl (6 mg/kg/h and 10 µg/kg/h, respectively). The ventilator strategy was 35-55 breaths min^−1^, FiO_2_ of 0.35-0.6 and a tidal volume of 9-11 ml kg^−1^. The use of high tidal volumes (as well as increasing respiratory rates) was a result of the attempt to compensate for the uncontrolled respiratory acidosis observed during the study. Since the increase in RR even when reaching maximal level did not suffice to decrease CO_2_, we chose to increase tidal volumes even though this entailed increasing minute ventilation. This relatively short period of several hours of potential volutrauma is not expected to contribute in a significant manner to the pathological findings described in the study. Positive end-expiratory pressure (PEEP) was set on the ventilator to 5-12, in agreement with the ARDS clinical guidelines.

### Physiological monitoring

#### Barometric plethysmography

Pigs were placed in a whole-body barometric plethysmograph (WBP, Buxco Electronics, NC, USA) and respiratory dynamics were measured before and at various time points after intoxication. The WBP was calibrated according to Goldman et al. (2005). The total volume of the WBP chamber was 400 liters and the airflow rate set for 500 l/min. A very small rejection index was used to correct for external noise. For measurement of respiratory parameters (Finepoint Software, Buxco Electronics, NC, USA), a baseline for each pig was recorded for 30 min following an acclimation period of 30 min in the chamber at each recording time point. The respiratory parameters monitored by WBP and their definitions are as follows: Respiratory rate (RR in rpm), instantaneous breath-by-breath rate of breathing; tidal volume (V_T_ in ml), the amount of air in a single breath; minute volume (MV in l/min), the product of V_T_ and RR calculated on a breath-by-breath basis; ratio of expiratory time (time spent exhaling during each breath) to inspiratory time (time spent inhaling during each breath, E:I); peak inspiratory flow (PIF in ml/s) and peak expiratory flow (PEF in ml/s), the maximum inspiratory and maximum expiratory flow that occurs in one breath, respectively; relaxation time (RT), the time it takes for the animal to expire 30% of volume; pause [(Te−RT)/RT], a rough calculation that correlates with bronchoconstriction; enhanced pause [PENH=(PEF/PIF)*×*pause], a rough estimate of lung resistance.

#### Cardiac output

Pigs were anesthetized before and 24 hpe to ricin by intramuscular injection of ketamine and xylazine (see above) followed by inhalation of 2-4% isoflurane with 100% oxygen. Measurements of non-invasive cardiac output (NICOM; Cheetah Medical, IN, USA) as well as invasive arterial blood pressure (IABP), 3-lead electrocardiogram and heart rate (Carescape Monitor B650, GE Healthcare) were taken. The bioreactance-based noninvasive cardiac output measurement system is based on an analysis of relative phase shifts of an oscillating current that occur when this current traverses the thoracic cavity. The NICOM system comprises: a radiofrequency generator for creating a high-frequency current that is injected across the thorax; four dual surface electrodes (placed at four corners of the thoracic body surface) that are used to establish electrical contact with the skin; and a receiving amplifier for recording the transthoracic voltage in response to the injected current. The signals from each side of the thorax are recorded separately and averaged after digital processing as described by Keren et al. (2007). The NICOM system has a pediatric calibration and therefore is validated for pigs at the same size and weight. Continuous IABP was measured by connecting the cannula, which was inserted into the left common carotid artery 3 days earlier (as described above), to a disposable pressure transducer (Biometrix, AS-0013). Air bubbles were flushed carefully from the system before data collection and zeroing was performed as instructed in the GE User Manual before data collection.

#### CXR

Chest X-ray images were obtained using a PXP-40HF machine (Poskom, Goyang, Korea) after inducing anesthesia by intramuscular injection of ketamine and xylazine, as above.

#### Blood and gas analysis

Arterial blood gas tensions, pH, hemoglobin and oxygen saturation were analyzed by an automated blood-gas and electrolyte analyzer (Osmetech OPTI CCA, Atlanta, GA, USA). White blood cells and neutrophil counts were determined by an automated hematology analyzer (Forcyte, Oxford Science Oxford, MS, USA).

### Lung permeability analyses

Pulmonary capillary leakage was determined by the Evans Blue dye (EBD) extravasation method as follows. EBD (2%, 20 mg/kg, Sigma) was injected into the ear vein, 15 min later animals were euthanized with sodium pentobarbitone (100 mg/kg, i.v.), the thorax was opened and the lungs and heart were removed following exsanguination by severing the major artery. To extract the dye, the excised lungs were immersed in formamide and incubated at 60°C for 24 h. The absorbance of the samples was measured at 620 nm in a spectrophotometer (Molecular Devices) and the total amount of dye was calculated by means of a standard calibration curve. To correct for contaminating heme pigments, extravasated EBD was calculated using the formula (Matute-Bello et al., 2011): E_620_(EBD)=E_620_−(1.426×E740+0.03). In other experiments, lungs were excised from naïve and ricin-intoxicated pigs and the ratio of lung/body weight (g/kg) was determined.

### Bronchoalveolar lavage (BAL)

BAL of pig lungs was performed before intoxication and 24 hpe by intratracheal insertion of a Metrix feeding tube (ConvaTec, Sunderland, UK) through a cuffed endotracheal tube (Well Lead, Guanguhou, China) and instillation of 30 ml PBS immediately thereafter recovering the fluid (∼20 ml) with a 50 ml syringe. Fluids were centrifuged at 3000 rpm at 4°C for 10 min, and supernatants were stored at −20°C until analysis. Cells from the BALF (1×10^5^) were resuspended in 100 µl PBS, precipitated onto glass slides (Cytospin 4 cytocentrifuge, Thermo Fisher Scientific, Waltham, MA, USA) and differential blood counts were determined after fixation with 70% ethanol and staining with Dip Quick Stain (Jorgensen Laboratories, Loveland, CO, USA) following the manufacturer's protocol. Concentrations of TNF-α, IL-1β and IL-6 were determined by porcine-specific enzyme-linked immunoabsorbent assays (ELISA, R&D systems, Minneapolis, MN, USA) according to the supplier's instructions. ET-1 concentrations were determined using a cross-reactive human ET-1 immunoassay commercial kit (R&D systems).

### Histology

Lung samples removed from sham or intoxicated pigs were fixed in neutral-buffered formalin, stained with H&E and examined by a pathologist. Five histological findings were graded using a semi-quantitative severity-based scoring system previously described (Matute-Bello et al., 2011). Briefly, values of 0-2 were used to score the severity of the following features: neutrophils in the alveolar and interstitial spaces, hyaline membranes, proteinaceous debris filling the airspaces and alveolar septal thickening, with 0 standing for no effect and 2 for maximum severity.

### Statistical analysis

Individual groups were compared using unpaired *t*-test analysis. To estimate *P* values, all statistical analyses were interpreted in a two-tailed manner. Values of *P*<0.05 were considered to be statistically significant.
